# Perceptual Grouping in Autism Spectrum Disorder: An Exploratory Magnetoencephalography Study

**DOI:** 10.1007/s10803-022-05844-0

**Published:** 2022-12-13

**Authors:** Christine M. Falter-Wagner, Christian M. Kiefer, Anthony J. Bailey, Kai Vogeley, Jürgen Dammers

**Affiliations:** 1 Department of Psychiatry, Medical Faculty, LMU Munich, Nussbaumstr.7, 80336 Munich, Germany; 2 Institute of Neuroscience and Medicine, INM-4, Forschungszentrum Jülich, Jülich, Germany; 3 Faculty of Mathematics, Computer Science and Natural Sciences, RWTH Aachen University, Aachen, Germany; 4 UBC Department of Psychiatry, University of British Columbia, 2255 Westbrook Mall, Vancouver, BC V6T 2A1 Canada; 5 Department of Psychiatry and Psychotherapy, University Hospital Cologne, Cologne, Germany; 6 Institute of Neurosciences and Medicine–Cognitive Neuroscience, INM-3, Forschungszentrum Jülich, Jülich, Germany

**Keywords:** Autism spectrum disorder (ASD), Gestalt perception, Grouping, Magnetoencephalography (MEG)

## Abstract

Visual information is organised according to visual grouping principles. In visual grouping tasks individuals with ASD have shown equivocal performance. We explored neural correlates of Gestalt grouping in individuals with and without ASD. Neuromagnetic activity of individuals with (15) and without (18) ASD was compared during a visual grouping task testing grouping by proximity versus similarity. Individuals without ASD showed stronger evoked responses with earlier peaks in response to both grouping types indicating an earlier neuronal differentiation between grouping principles in individuals without ASD. In contrast, individuals with ASD showed particularly prolonged processing of grouping by similarity suggesting a high demand of neural resources. The neuronal processing differences found could explain less efficient grouping performance observed behaviourally in ASD.

## Introduction

Besides impairments in social interaction and communication as well as restricted and stereotyped interests and behaviours (APA, 2013), individuals with autism spectrum disorder (ASD) also show a range of unusual visual processes (e.g., Dakin & Frith, 2006). In particular, superior performance in some visuo-spatial tasks, such as Block Design and Figure-Disembedding (for a meta-analysis see Muth et al., 2014) have led to the assumption of a more detail-focussed processing style (Fitch et al., 2015; Frith & Happe, 2006), with reduced or spared global processing capacities depending on task requirements and instructions (Koldewyn et al., 2013; Mottron et al., 1999; O’Riordan & Plaisted, 2001; O’Riordan et al., 2001; Plaisted et al., 1999).

In general, visually presented information is organised according to classical Gestalt laws (e.g., Koffka, 1935), such as proximity (i.e. objects are grouped according to spatial closeness) or similarity (i.e. objects are grouped according to shared features). In an early classification of visual processing, a division into so-called type P and type N relationships was suggested (Pomerantz, 1983): While type P relationships are characterised by the placing of elements (e.g., closeness), type N relationships are characterised by the nature of visual elements (e.g., similar appearance). Individuals with ASD show particular weakness in the perception of type N relationships (e.g., Brosnan et al., 2004; Falter et al., 2010).

Nevertheless, whether visual grouping processes can be considered typical or atypical in ASD is still not settled. Studies employing tasks that explicitly measure grouping processes on the basis of participants’ introspection, showed impairments in perceptual grouping in ASD, particularly with respect to grouping by similarity (type N; e.g., Boelte et al., 2007; Brosnan et al., 2004). Similarly, a study measuring grouping by five different Gestalt principles showed weaker perception of grouping by similarity (type N) with respect to shape, orientation and luminance, and spared grouping by proximity (type P) and alignment (Farran & Brosnan, 2011).

The introduction of object-based attention tasks (Feldman, 2007) allowing the implicit measurement of how grouping principles influence perceptual judgments, showed a differentiated picture of impaired grouping by colour similarity (type N) and intact grouping by proximity (type P) in ASD (Falter et al., 2010). Perceptual grouping in ASD also seems to depend strongly on general task requirements and instructions. For instance, symmetry perception in individuals with ASD has been found to be both superior (Perreault et al., 2011) and inferior (Falter & Bailey, 2011), depending on task design and underlying constructs, with the authors of the two studies drawing contradicting conclusions (for a discussion see Falter, 2012). Thus, task design and instructions might play a role in shaping participants’ performance in grouping tasks. Similarly, sampling and inclusion criteria that vary between studies might likewise cause group differences to be found in some studies and not in others. Recent studies measuring grouping processes *implicitly* in ASD showed typical influence of four different, both type P and N, grouping principles (similarity, proximity, closure, and good continuation) on stimulus distance estimations in ASD (Avraam et al., 2019) and typical shape formation depending on grouping cues, such as closure, proximity and collinearity (Hadad et al., 2019). Likewise, grouping of distractors was found to be typical in a visual search paradigm in individuals with ASD (Keehn & Joseph, 2016) and visual speed discrimination thresholds were equally strongly influenced by grouping of stimuli in children with and without ASD (Manning & Pellicano, 2015). These series of implicit measures therefore suggest typical grouping perception in ASD and a lack of impairments. Yet, such a conclusion could be premature: Even implicit measures of perceptual grouping processes show inconsistent results of reduced (Evers et al., 2014) or typical grouping interference on multiple object tracking abilities in children with ASD (Van der Hallen et al., 2018), again depending on exact task requirements and study design (e.g., ratio of grouped versus ungrouped stimuli; for a discussion see Van der Hallen et al., 2018). Therefore, an answer to the question whether perceptual grouping is atypical or typical in ASD remains unsettled. Thus, in this study we turned our attention to the neural processing mechanisms of grouping principles in ASD. The rationale behind this approach is that, irrespective of typical or atypical performance levels in grouping tasks, we do not know whether the underlying neural mechanisms recruited by individuals with ASD resemble those used by typically-developing (TD) individuals.

Studies on neural processing of visual grouping in TD individuals found that grouping happens early in the visual processing stream and that different Gestalt laws appear to correspond to different neural mechanisms (Han et al., 2002). In an event-related potential (ERP) study, grouping by the law of proximity (type P) has been shown to be associated with short-latency positivity over the medial occipital cortex followed by a right occipitoparietal negativity, whereas grouping by shape similarity (type N) elicited a long-latency occipitotemporal negativity (Han et al., 2001). Similarly, grouping by colour similarity (type N) was associated with long-latency occipito-temporal modulations (Han et al., 2002). These ERP-findings suggest that distinct neural substrates are associated with visual organisational processes based on different Gestalt laws in TD participants.

Although there are no neuroimaging studies investigating mechanisms of Gestalt grouping in ASD, there are suggestions of different processing strategies for Gestalt grouping. Farran and Brosnan investigated grouping by shape similarity (type N) with varying difficulty levels across stimulus displays. They found that when participants with ASD employed different processing strategies compared to TD controls, they yielded a similar accuracy. However, when they relied on the same strategies as TD controls, their performance was impaired (Farran & Brosnan, 2011).

The aim of the current study was to compare neural correlates of Gestalt grouping in individuals with and without ASD. Measuring neuromagnetic activity, participants were presented with two of the most commonly tested Gestalt grouping principles, proximity (type P) and similarity (type N), in the well-established paradigm used by Han and colleagues (Han et al., 2001, 2002). Across groups, we expected grouping by proximity to be processed at an earlier and grouping by similarity at a later stage (Quinlan & Wilton, 1998). Furthermore, on the basis of previous behavioural results, we expected deviant patterns of processing Gestalt grouping principles in individuals with ASD compared to TD individuals.

## Methods

### Participants

Nineteen participants with ASD were recruited through a database at the Department of Psychiatry, University of Oxford. They had a diagnosis of either high-functioning autism or Asperger syndrome according to DSM-IV-TR (APA, 2013). Diagnoses were confirmed by the Autism Diagnostic Interview–Revised, ADI-R (Lord et al., 1994) and the Autism Diagnostic Observation Schedule–Generic, ADOS-G (Lord et al., 2000). The ADI-R age of onset criterion was not met by two participants and three participants scored one point below threshold on one ADI algorithm domain. Overall, all these participants scored above the ASD cut-off on other ADI domains and were included in the final analysis. Exclusion criteria for ASD participants were comorbid psychiatric disorders, psychotropic medication, and a Wechsler Abbreviated Scale of Intelligence (Wechsler, 1999) full scale IQ < 85. Three participants with ASD showed performance at a level of at least 2 standard deviations below the group mean and their data were excluded from further analysis. Data of another participant from the same group was excluded due to severe movement artefacts.

Nineteen TD control participants were recruited through local advertisements. In addition to the described exclusion criteria for the ASD group, TD participants were only included if they were free of any psychiatric diagnoses. All individuals taking part in the study had normal or corrected-to-normal vision; colour-blindness was excluded using the Ishihara Colour Blindness Test (Ishihara, 1971). One TD participant did not complete all the assessments and was therefore not included in the final sample.

The final sample consisted of 15 participants with ASD and 18 TD participants. The two groups (see Table 1 for demographic data) were matched on age, verbal IQ, and performance IQ (largest *t* = 1.30). Informed consent was obtained from all participants prior to any testing and the study received ethical approval from the NHS Newcastle & North Tyneside 2 Research Ethics Committee (Newcastle 2 REC 08/H0907/66).

**Table 1:** Demographic data

	ASD (*n* = 15; 1 female)			TD (*n* = 18; 3 females)		
	Mean	SD	Range	Mean	SD	Range
Age	26:10	9:8	16:9–44:0	26:6	6:5	15:9–38:6
VIQ	110	15	70–125	114	12	99–139
PIQ	114	11	92–136	116	9	104–136
FIQ	113	11	89–130	116	9	101–141
ADI-A	18	5	12–28			
ADI-B	15	4	9–21			
ADI-C	6	3	2–12			
ADOS-A	3	2	1–6			
ADOS-B	6	3	1–12			
ADOS-C	1	1	0–3			

Means, standard deviations, and ranges of age (years:months), verbal intelligence quotient (VIQ), performance IQ (PIQ), and full IQ (FIQ) of participants with autism spectrum disorder (ASD) and typically-developing (TD) participants. Autism Diagnostic Interview-Revised (ADI-R) Social Interaction Domain (ADI-A), ADI-R Communication Domain (ADI-B), ADI-R Repetitive Behaviours Domain (ADI-C), Autism Diagnostic Observation Scale-Generic (ADOS-G) Communication Domain (ADOS-A), ADOS-G Reciprocal Social Interaction Domain (ADOS-B), and ADOS-G Stereotyped Behaviours and Restricted Interests Domain (ADOS-C) of participants with ASD

### Design and Procedure

**Fig. 1:**
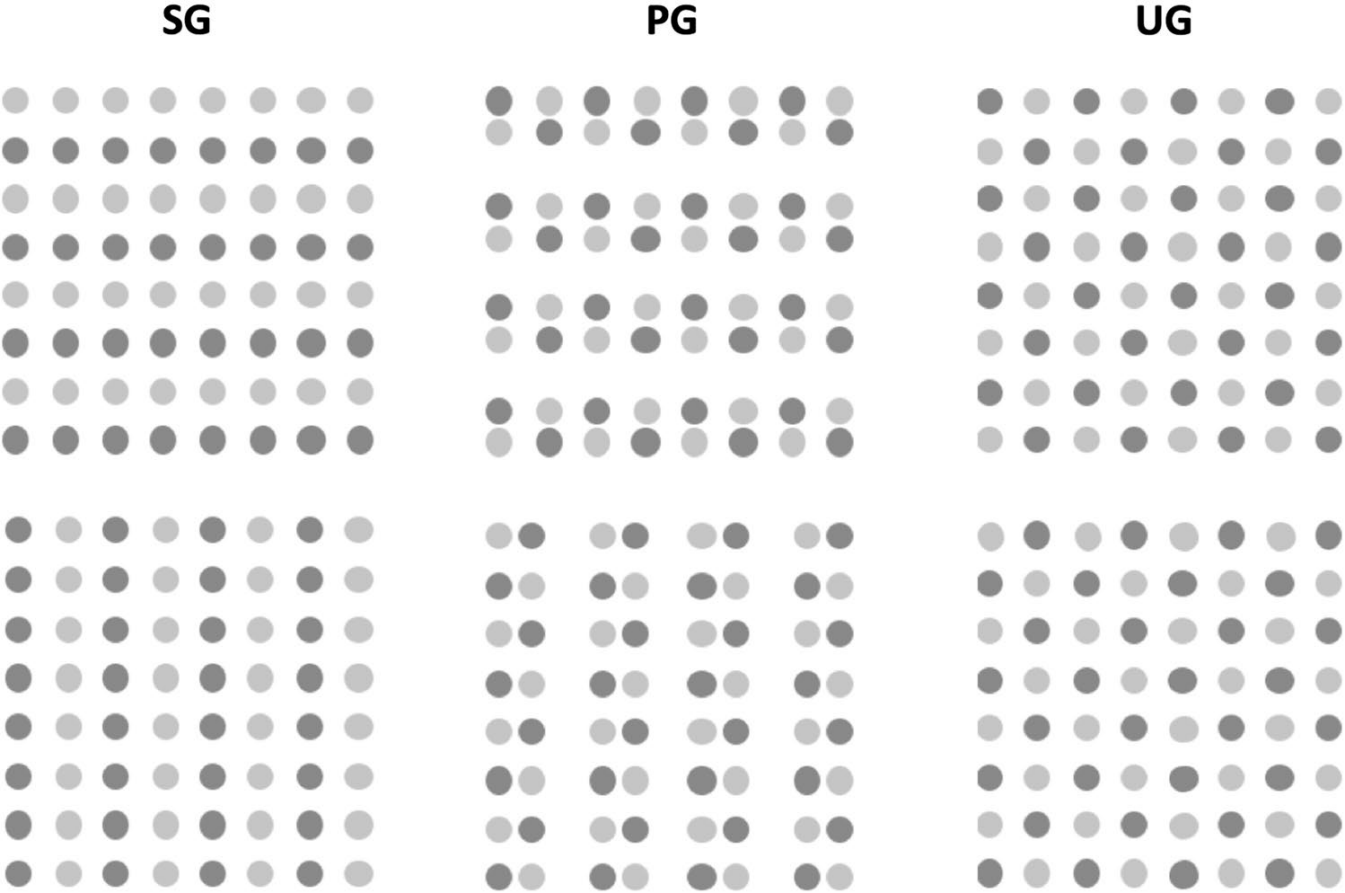
Types of stimuli used in the grouping task (note that circle elements were blue and red in the original task). In the similarity grouping condition (SG), the circle elements are organised by colour to elicit similarity grouping. In the proximity grouping condition (PG), the circle elements are organised by proximity between different coloured circles to elicit proximity grouping. In the control condition, termed uniform grouping (UG), the circle elements are distributed uniformly without eliciting perceptual grouping

Stimuli were presented using the software Inquisit (Draine, 2003) and were back-projected onto a translucent screen in a dimly lit scanning room (0.1 cd/m^2^). The experiment and stimuli followed the design of Han and colleagues (Han et al., 2002). Participants were presented with square lattices of 8 × 8 blue and red circle elements (0.47° per element; 7.8° per lattice; see Fig. 1) on a black screen (0.02 cd/m^2^). The elements were either distributed with the same colours forming rows or columns (similarity grouping, SG, type N) or with pairs of red and blue circle elements forming rows or columns (proximity grouping, PG, type P). According to Han et al. (2002), a control condition with elements uniformly distributed was included so that participants were required to pay attention to the type of grouping to make their decision. Participants were given one left and one right hand-held light-sensitive response button and were instructed to press the left or right key using their thumbs if the presented circles were organised into rows or columns, respectively. In case no decision could be made (i.e. uniform distribution in the control condition), both keys should be pressed.

In each trial participants were asked to fixate a white cross (0.3° × 0.2°) of 1000 ms duration, followed by the stimulus presentation of 200 ms duration and a blank screen until a response prompt was presented after 1300 ms. Following the participant’s response, a random inter-trial interval of 800–1200 ms preceded the next trial. Grouping conditions (SG, PG, control condition) were presented in 300 trials (i.e. 100 trials each) with rows and columns conditions equally counterbalanced and distributed in random order. After a training block of about 10 trials the experiment was performed in 4 blocks of 75 trials each. Participants’ understanding of the instructions was confirmed before starting the experiment.

#### Data Acquisition and Preprocessing

Magnetoencephalography (MEG) data were continuously acquired using a Neuromag-306 VectorView™ system housed in the Oxford Neurodevelopmental Magnetoencephalographic Unit; this consisted of a helmet-shaped array of 102 pairs of orthogonal, first-order planar gradiometers and 102 magnetometers (306 channels in total). The data were sampled at 1000 Hz and online filtered from 0.03 to 330 Hz using an anti-aliasing filter during acquisition. Individual head position was measured before each experimental run. Eye movements and cardiac activity were recorded in synchrony with the MEG signals using electrooculography (EOG) and electrocardiography (ECG).

Signal-space separation (SSS) was applied to the MEG raw data to suppress magnetic noise interference originating from outside the helmet (Taulu & Simola, 2006). Data were further processed and analysed using the MNE-Python toolbox (Gramfort et al., 2014). An offline bandpass filter was applied with cut-off frequencies at 1 and 45 Hz and notch filters around the powerline frequency and harmonics. Biological artefacts were identified and removed using independent component analysis (ICA) as implemented in MNE-Python. Components related to ocular or cardiac activity were identified using correlation and cross-trial phase statistics, CTPS (Dammers et al., 2008), as implemented in MNE-Python.

Epochs were constructed around stimulus onset (200 ms pre- and 600 ms post-stimulus) using the epochs of correct responses only. The trial distribution in each group was equalised to match mean and variance. For each condition and group, the average signal strength from the 200 ms pre-stimulus interval was used for baseline correction.

#### Source Activity Estimation and Region of Interest Analysis

Anatomical MRI scans were only available for a subgroup of participants (ASD: *n* = 6, TD: *n* = 7). T1-weighted structural scans were acquired (at FMRIB, Oxford) with a 1.5 T clinical scanner (Siemens) with the following parameters: repetition time = 12 ms, echo time = 5.65 ms, flip angle = 19 degrees, slice thickness = 1 mm, in-plane resolution = 1 mm^2^. For segmentation of the brain the FreeSurfer software package was used (Dale et al., 1999; Fischl et al., 1999, 2001). For the participants (ASD: *n* = 9, TD: *n* = 11) for whom individual MR scans were not available, the ‘FsAverage’ template, an average participant scan provided by FreeSurfer, was used as a substitute (Fischl et al., 2001).

To map the measured neuromagnetic activity onto brain anatomy the open source MNE-Python toolbox was used (Gramfort et al., 2014). Transformation matrices for the alignment of the MR-coordinate system and the head-coordinate system of the neuromagnetic data were computed for participants with MR scans. For participants without individual MR scans the digitised head shape was used for registration onto the FsAverage MR template. Manual adjustment and visual inspection were performed using the graphical user interface of the coregistration tool as implemented in MNE-Python. All other subsequent analysis steps were identical for all participants.

To verify the validity of the coregistration using the template brain instead of an individual anatomical MRI, the coregistration was performed on the FsAverage template for all participants with and without existing MR scans. Thus, two source spaces (individual MRI, template MRI) were obtained for comparison in all those participants for whom MR measurements were obtained. After transforming both source spaces into the same coordinate system, 68 locations of the centres of masses of the anatomical regions defined by the Desikan-Killiany atlas were compared (Desikan et al., 2006). An average deviation of 1.15 cm (± 0.24 cm) across participants confirmed that the localisation error introduced by using the template brain was at an acceptable level and substantially smaller than the size of the anatomical regions defined for the purpose of this study, namely, cortical areas with at least 7 cm^2^ and a radius of at least 1.5 cm (see below).

The forward problem was solved using the boundary element method (BEM) with 5124 vertices as implemented in MNE-Python, which resulted in an average grid spacing of 6.2 mm. For source analysis, dynamic statistical parametric mapping (dSPM) (Dale et al., 2000) was applied with a default depth weighting of 0.8 (Lin et al., 2006). The estimated cortical activations were morphed to the FsAverage standard brain (Dale et al., 1999; Fischl et al., 1999).

Regions of interest (ROI) were determined for each grouping condition from averaged source activity. A Monte-Carlo-based non-parametric spatio-temporal cluster permutation test was performed to extract within and between group-based statistical maps of distinct spatio-temporal clusters (Maris & Oostenveld, 2007) with sustained (≥ 20 ms) temporal activations (Larson & Lee, 2013, 2014; Maris & Oostenveld, 2007). To identify differences across conditions within the participant groups, a two-sample permutation t-test with 10^4^ permutations was performed between the conditions SG and PG for the ASD and control group separately. As a cluster threshold criterion, we used a critical alpha level of 0.01. The significance level for the permutation test was set to p < 0.05. As the cluster level permutation test addresses the multiple comparison problem (MCP), the differences across conditions directly reflect the significance level at the same time (Maris & Oostenveld, 2007).

In addition, differences between participant groups were investigated using a two-sample permutation independent t-test with the same parameter as described above (10^4^ permutations, cluster threshold with a critical alpha level of 0.01, p < 0.05).

To align the cluster activity onto brain structure, we used the Desikan-Killiany atlas providing 68 cortical parcellations (Desikan et al., 2006). After delineation, small clusters, i.e., a cluster with fewer than 19 vertices (corresponding approximately to a neuromagnetically active concentric area with a radius of less than 1.5 cm or an active cortical area of 7 cm^2^) or durations shorter than 20 ms, were discarded from analysis. The remaining clusters were used as ROIs in the following analyses.

#### Time Course Analysis

Representative source time courses (rSTC) were constructed for each ROI using the STC extraction tool provided by MNE-Python (Gramfort et al., 2014). The mean of all vertex activations, as defined by the ROI, was computed and used for further analyses. Finally, for subsequent statistical analyses, all rSTCs were z-scored using the mean and standard deviation from the pre-stimulus interval (200 ms prior to stimulus onset) of each ROI and across all trials.

Cluster permutation tests were applied (Maris & Oostenveld, 2007) to all rSTCs using an F-test (10^4^ permutations, cluster threshold with a critical alpha level of 0.05) to extract between participant groups differences in the temporal dynamics of the identified ROI.

### Community Involvement Statement

Community members were not involved in the study.

## Results

### Behavioural Performance

There was a slight difference in sex ratio (3 TD females, 1 ASD female) between the two groups, but no differences in performance between female and male participants and consequently data from both sexes were combined.

A mixed ANOVA with one between-participants factor of Group (ASD vs. TD) and two within-participants factors of Gestalt (PG vs. SG) and Orientation (Rows vs. Columns) was performed on accuracy data. The analysis showed a main effect of Group (*F*(1, 31) = 6.143, *p* = .019, partial η^2^ = .165) indicating that the TD group performed overall more accurately than the ASD group, albeit both groups performed at comparably high performance levels close to ceiling (TD: PG Mean = 98.0 ± 2.1, SG Mean = 97.9 ± 2.0; ASD: PG Mean = 95.4 ± 5.1, SG Mean = 94.8 ± 5.6). There were no other main effects or interactions between the three factors (all *F* < 1.09).

### Spatio-temporal ROI Analysis

The number of trials used for averaging and subsequent source analysis was 93.5 (± 5.9) on average after excluding incorrect trials and equalisation of the trial distribution between participant groups. Spatio-temporal cluster permutation analysis was applied to explore both differences between grouping types and differences between participant groups.

#### Within-Participant Analysis of Neuromagnetic Activation of Different Grouping Types

**Fig. 2:**
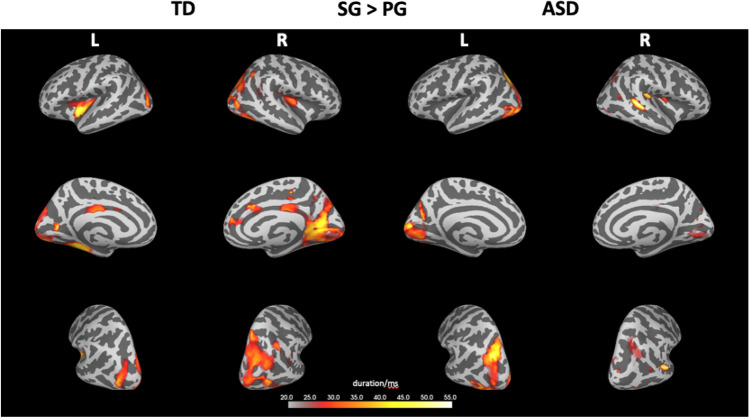
Spatio-temporal cluster of significantly different source activations for the contrast (SG–PG) within participants, separately for both diagnostic groups. Neuromagnetic activity was always found to be significantly stronger during SG in both participant groups. The colour bar indicates the duration (in ms) where the activity of SG was found to be larger (p < 0.05). L and R specify the side of the hemisphere

In both groups the neuromagnetic activations during SG were generally found to be stronger than those during PG (Fig. 2). Furthermore, the TD group showed more distributed activity with higher hemispheric lateralisation (Fig. 2). In particular, the two-tailed cluster permutation test revealed 3 clusters in the TD group and 2 clusters in the ASD group with stronger activity during SG. In both groups, no cluster of activity was found to be stronger during PG in contrast to SG. The temporal extent of all clusters ranged from 83 to 173 ms post stimulus with an average duration of 64.4 ms and a stronger lateralisation towards the right hemisphere in the TD group (Fig. 2).

### Between-Participant Analysis of Neuromagnetic Activation

**Fig. 3:**
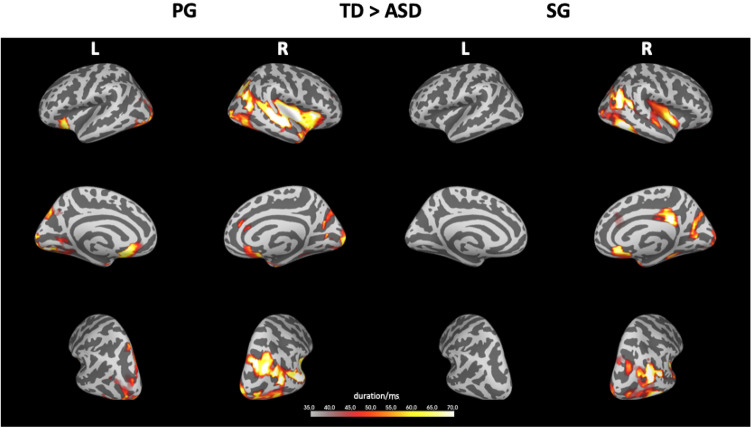
Between-participants differences during different grouping types PG and SG. The activity was found to be larger in the TD group. The colour bar indicates the duration (in ms) of the difference in source activity (p < 0.05)

To explore the spatio-temporal processing of the two grouping conditions between participant groups, we were interested in comparing the source activity in different time windows and the two grouping conditions. Spatio-temporal cluster permutation tests using a two-tailed independent t-test were applied to extract differences between the two participant groups. A direct comparison of spatio-temporal cluster activations between groups revealed that during processing of PG and SG, the neuromagnetic activity was larger in the TD group in both conditions. In particular, 3 clusters with significant differences (p < 0.05) in their activation strength were found (Fig. 3). The temporal extent of all clusters was found to be in the time window ranging from 0 to 244 ms post-stimulus with an average duration of 166 ms.

**Fig. 4:**
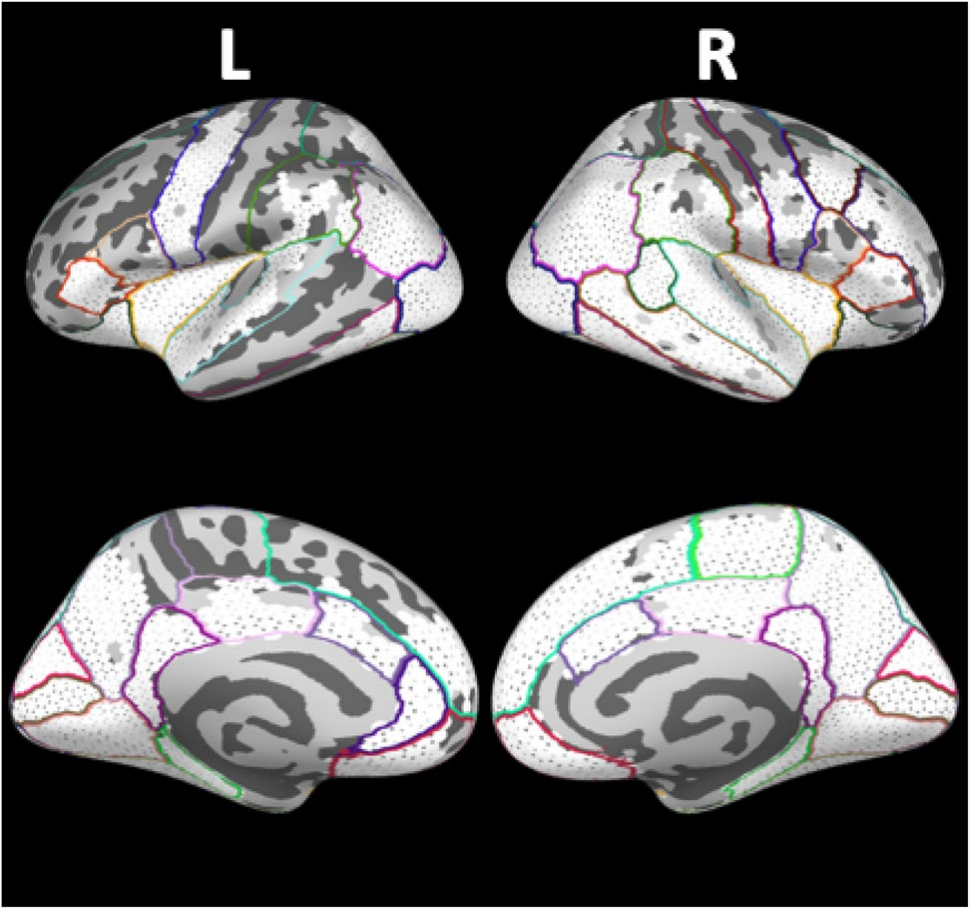
Spatial maps of all anatomical regions with combined clusters of significantly different neuromagnetic activity either between groups and/or between conditions. Vertices of clusters are shown in white. Boundaries of regions according to the Desikan-Killiany atlas are highlighted in different colours

For subsequent analyses of the temporal dynamics, all clusters of significantly different brain activation, either between groups and/or between conditions, were integrated into one set of potentially informative brain regions. In total, spatio-temporal statistical testing revealed differences in brain activity distributed over 23 brain regions in the left hemisphere and 28 regions located in the right hemisphere, respectively. The corresponding anatomical regions are displayed in Fig. 4 and are summarised in Table 2.

**Table 2:** Regions of interest

ROI	Anatomical label	LH	RH
BSTS	Banks of superior temporal sulcus		X
ACC	Caudal anterior cingulate cortex	X	X
MFC	Caudal middle frontal cortex		X
Cuneus	Cuneus	X	X
FG	Fusiform gyrus	X	X
IPC	Inferior parietal cortex	X	X
ITG	Inferior temporal gyrus	X	X
Insula	Insula	X	X
ICC	Isthmus cingulate cortex	X	X
LOC	Lateral occipital cortex	X	X
LOFC	Lateral orbitofrontal cortex	X	X
LG	Lingual gyrus	X	X
OFC	Medial orbitofrontal cortex	X	X
MTG	Middle temporal gyrus		X
Para	Paracentral region		X
PPA	Parahippocampal place area	X	X
POC	Pars opercularis	X	X
PT	Pars triangularis	X	X
PeriCC	Pericalcarine cortex	X	X
PoC	Postcentral cortex		X
PCC	Posterior cingulate cortex	X	X
PrC	Precentral cortex	X	X
PreCC	Precuneus	X	X
rACC	Rostral anterior cingulate cortex	X	
rMFC	Rostral middle frontal cortex		X
SFC	Superior frontal cortex	X	X
SPC	Superior parietal cortex	X	X
STG	Superior temporal gyrus	X	X
SMG	Supramarginal gyrus	X	X

Regions of interest in the left (LH) and right hemisphere (RH). In total 23 and 28 ROIs with significant differences (X) were identified in LH and RH, respectively

### Time Course Analysis

Time course analysis was performed on all identified ROIs (cf. Table 2) and revealed differences between conditions mainly during the first evoked response (80–160 ms post-stimulus) in both groups. Statistical analysis between different grouping types confirmed that the early activity (0-200 ms) during PG was always found to be smallest.

When comparing the temporal dynamics between participants, the strength of activity was found to be larger in the TD group in both conditions during early processing. In particular, significant differences in the evoked responses were observed in 7 ROIs in the left (3 for SG, 4 for PG) and 27 (14 for SG, 13 for PG) ROIs in the right hemisphere, respectively.

When comparing the early responses between groups in the time window ranging from 0 to 200 ms, a significantly later peak latency of the evoked response was found in the ASD group as compared to the TD group in both grouping conditions (*n* = 16 for SG and *n* = 14 for PG; Table 3).

**Table 3:** Peak latencies (means and standard deviations, SD) in milliseconds in the conditions similarity grouping (SG) and proximity grouping (PG) and temporal differences (Δt) with significance levels between grouping conditions

		ASD	TD	Δt	*p*
SG	Mean	132.8	123.9	8.8	0.00001
	SD	13.4	13.6		
PG	Mean	135.2	127.8	7.5	0.00115
	SD	13.8	12.8		

**Fig. 5:**
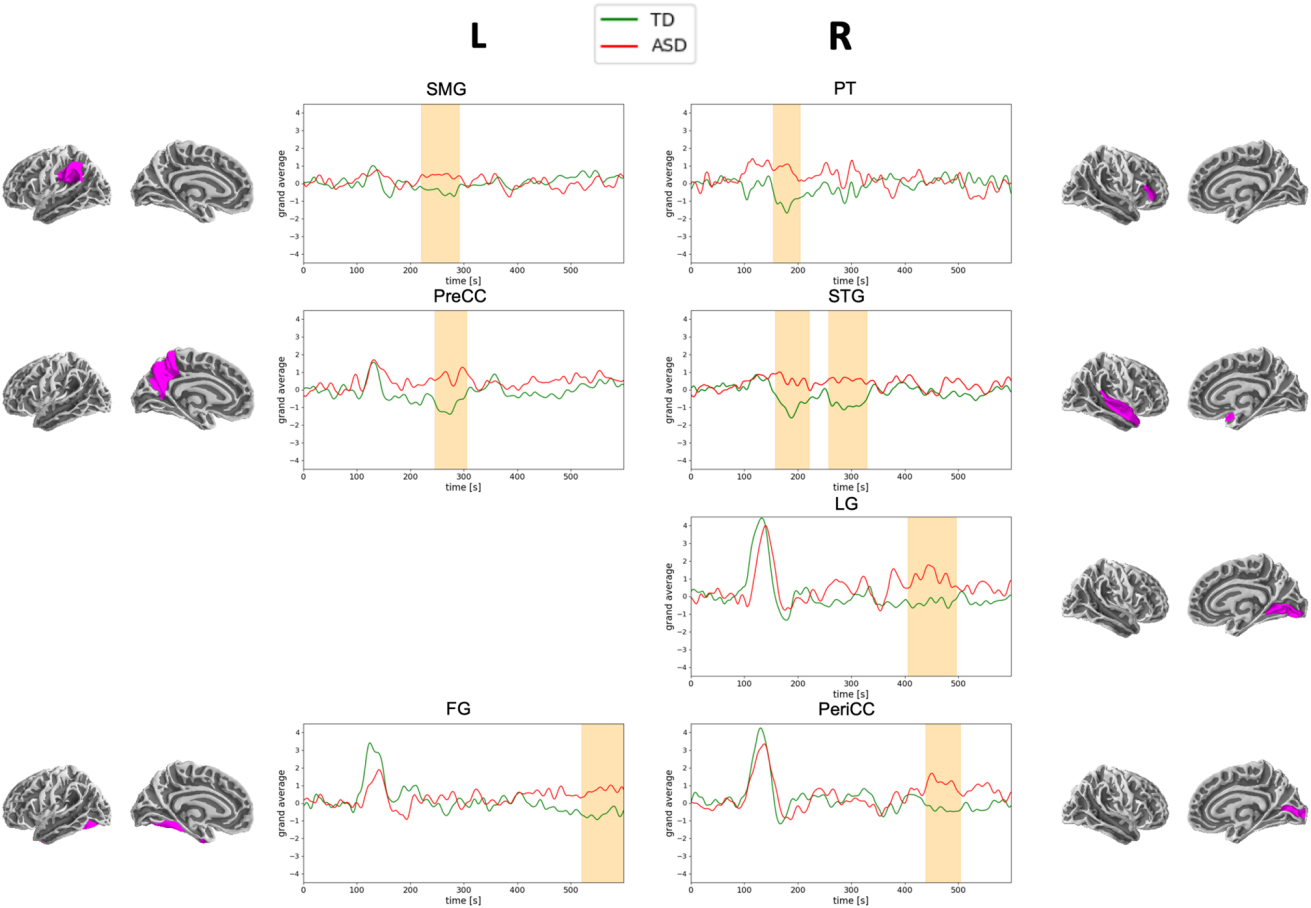
Temporal dynamics of the contrast SG-PG in ASD (red) and TD (green). Time windows of significantly different activation profiles are highlighted in orange. Activation above zero indicates stronger activity during SG. Corresponding anatomical areas are shown for the left (LH) and right hemisphere (RH)

To elucidate more subtle differences in the processing of visual grouping, a permutation cluster test on ROI time courses was applied on the contrast of grouping type [SG-PG]. Differences in the contrasted activity and between the two groups were found in 3 regions of the left (SMG, PreCC, and FG) and in 4 regions of the right hemisphere (PT, STG, LG, and PeriCC) (Fig. 5). The neural activation was stronger in ASD in all later time windows showing significant results between contrasts and groups.

## Discussion

The aim of the current study was to explore the neural mechanisms underlying classic Gestalt grouping processing in visual displays in individuals with ASD using neuromagnetic activity. The main findings are summarised as follows: (i) stronger neuromagnetic activity during similarity grouping (SG) compared to proximity grouping (PG) in both participant groups, possibly suggesting lower processing demands during PG (see Fig. 2), which might be more readily discernible and differentiable compared to SG in both groups; (ii) significantly reduced and slower activity in the ASD group compared to the TD group within the first 200 ms after stimulus onset, irrespective of the grouping condition; (iii) a complex set of interactions between group and condition was found in different brain regions, with higher activities at later latencies (Fig. 5) in occipital and superior parietal areas in ASD and a more distributed activity in the right hemisphere in the TD group (see Fig. 3).

Against the backdrop of persistent inconsistencies in the findings of behavioural performance of individuals with ASD in Gestalt grouping tasks, as outlined in the introduction, the use of different neural strategies for Gestalt processing has been discussed (Farran & Brosnan, 2011). The rationale for using a direct measure in this MEG study was to explore the neural correlates of grouping in a categorical design. For this purpose, we chose a very simple Gestalt grouping design established before (Han et al., 2001, 2002), which yielded in our study above 94% correct answers in both participant groups, allowing us to investigate any differences in neural mechanisms underlying Gestalt grouping processes during low task demands.

In both groups neuromagnetic activations during SG were stronger than during PG. Yet, despite this similarity between groups, we also found significantly higher and earlier amplitudes in the TD group during both types of grouping (SG and PG) as compared to the ASD group. Hence, despite the speculatively higher processing demand of SG over PG in both participant groups, the TD group seems to be better prepared for this task. The delayed processing of grouping information in ASD could be in accordance with previous reports of a sluggish cognitive tempo in ASD (e.g., Brewe et al., 2020).

Comparing localisation of evoked activity, the contrast between SG and PG was found to be significantly different between groups in seven different ROIs. Two superior parietal areas (PreCC, SMG), three visual areas (FG, LG, PeriCC), one temporal area (STG), and one frontal area (PT) showed stronger activity at later latencies in ASD compared to TD (Fig. 5). This difference could be interpreted as high demand of SG as opposed to PG requiring increased neural activation in brain regions responsible for the processing of visuo-spatial material in persons with ASD. This specific information about grouping processing could explain why the performance in response to PG is faster and easier compared to SG (e.g., Falter et al., 2010; Farran & Brosnan, 2011). Similarly, increased reliance on neural processing of visuo-spatial information in posterior regions in individuals with ASD has been reported before (e.g., Falter, 2012; Kumar, 2013).

A second neuroanatomical observation was the more distributed and stronger activation in the right hemisphere of TD persons, when compared to the ASD group. Thus, it seems that the TD group showed a more pronounced hemispheric specialisation (e.g., Allen, 1983), putatively enabling a more efficient resolution of perceptual demands. Our results corroborate several previous reports of reduced or atypical hemispheric asymmetry in ASD. For instance, significantly reduced white matter microstructure asymmetry has been found in ASD (Carper et al., 2016) and atypical asymmetry patterns of activity were found in a previous MEG study on sentence reading (Ahtam et al., 2020). Many previous findings relate atypical hemispheric asymmetries to language impairment in ASD (Lindell & Hudry, 2013) but several functional networks have recently been found to show atypical hemispheric asymmetry beyond language functionality (Cardinale et al., 2013). Alternatively, less hemispheric asymmetry might be due to unrelated or noisy activity in one hemisphere in ASD.

Our interpretation of stronger specialisation in the allocation of neuronal processing in the TD group according to task requirements (Fig. 3), compared to a less specialised response in the ASD group, might be a general characteristic of the autistic neuro-cognitive profile in that individuals with ASD might adjust processing less specifically to conditions. Indeed, we previously also found less specific neural activity to task-specific requirements for auditory duration versus pitch perception in ASD compared to a matched TD group (Lambrechts et al., 2018).

Particularly interesting is the significantly increased precuneus activity at 220–300 ms found for SG in contrast to PG in the ASD group only. Increased precuneus activation has previously been found in individuals with ASD during sustained attention: in the ASD group activation was found to progressively increase in the precuneus with increasing sustained attention load, in contrast to progressively decreasing activity found in the control group, which the authors interpreted as difficulty with default mode network suppression in ASD (Christakou et al., 2013). Furthermore, increased precuneus activation was positively correlated with social symptom severity as measured with the ADOS (Christakou et al., 2013). This finding again supports the proposal of a higher cognitive load during SG as opposed to PG that is specific to persons with ASD.

Although not directly comparable, our findings are generally speaking in line with studies of Gestalt grouping using fMRI. A recent fMRI study on spontaneous Gestalt processing showed particular activity in the superior parietal lobe and the anterior intraparietal sulcus associated with grouped illusory Gestalt perception (Zaretskaya et al., 2013). Likewise, the regions of interest found in the current study are in keeping with the report of intact versus disturbed global Gestalt perception in hierarchical stimuli (i.e. global shapes made of local elements) leading to activity in precuneus, temporo-parietal junction, and anterior cingulate cortex (Huberle & Karnath, 2012). In addition, Han et al. (2005) showed proximity grouping associated with calcarine cortex, inferior parietal cortex (LH and RH) and right superior temporal cortex. Similarity grouping was associated with right middle occipital cortex, left middle temporal cortex, yet similarity was based on shape similarity, not colour similarity. Thus, an overlap of regions found responsible for Gestalt grouping in fMRI studies and the sources located in the current MEG study can be asserted. Employing MEG we could additionally carve out slower activity in the ASD group compared to TD controls.

Behaviourally, we observed a high accuracy with more than 94% correct answers, irrespective of diagnostic group or grouping process studied. Despite of the statistically significant difference between diagnostic groups, accuracy scores clearly showed a ceiling effect in an obviously low cost task; in addition, the percentage of incorrect trials, which were below 5% of all trials (ASD: 4.9%; TD: 2.1%), was very low so that we considered the direct comparison of the neural correlates of correct trials in the different conditions across both groups as justified. The observation of very high accuracy scores in both conditions and among both diagnostic groups of at least 94%, might be due to our design that directly assessed grouping in a categorical design, rather than using a paradigm indirectly measuring grouping strength in a parametric design. Discrepancies in findings of intact or impaired performance in tasks employing Gestalt principles in ASD might be due to direct versus indirect measurement (Farran & Brosnan, 2011). Future studies should focus on “critical” tasks that have been shown in the past to lead to group-difference results–in comparison to tasks that have been shown to show equivalent performance—in the same sample.

A limitation of our study was that only a part of our participants agreed to have their MRI scans taken (n = 13). Instead of excluding all participants for whom MRI scans were not available and to avoid the risk of having less representative sample sizes, we decided to include theses data into the analysis. For these participants, a template scan (as a substitute for an individual MRI) was used as provided by FreeSurfer package (Fischl et al., 2001). The average localisation error (distances between the centre of masses of correctly coregistered areas using the individual MRI and a registration based on a template) due to missing MRIs was small (estimated at 1.15 cm). Our ROI definition was based on relatively large regions as defined by the Desikan-Killiany atlas. The mean surface area of our ROIs defined in Table 2 was estimated at about 30.70 cm^2^, which would result in a diameter of about 6.26 cm for circular areas. In other words, a displacement of the centre of mass of significantly active vertices in the range of the coregistration error is very likely to be located in the same anatomical area.

A limiting factor of a priori hypothesis setting was the state of art of neuroscientific (in particular MEG) data on Gestalt processing at the time of study design. The current study should be considered exploratory. Given the limited sample size included and the unconstrained analysis, the interpretation of results should be treated with caution and submitted to scientific scrutiny in future research studies Thus, there remain several potential explanations for the group differences in MEG findings. We cannot entirely rule out that (i) these could be based on chance, (ii) the group differences might indicate different processing strategies related to slightly decreased performance in the ASD group, or (iii) the group differences might reflect compensatory processing in the ASD group. Finally, concerning any group differences in recordings of brain activity one cannot entirely rule out that these may be due to uncontrollable confounds that are not accessible by any behavioural assessment.

## Conclusion

Taking the results together, we found stronger evoked responses with earlier peaks in response to both types of grouping in the TD group compared to the ASD group. First, there might be an earlier differentiation between SG and PG in the TD group with individuals with ASD showing particularly prolonged processing of SG. Second, grouping by similarity might pose a particularly high demand on neural processing for individuals with ASD and the processing in the ASD group might be characterised by a prolonged demand of high neuronal processing for similarity grouping. This high demand is potentially due to a decreased hemispheric specialisation in ASD as indicated by the stronger hemispheric asymmetry in TD suggesting more specialised and focused neural mechanisms. Additionally, our results are in line with the idea of reduced default mode suppression in ASD. Speculatively, our results suggest that Gestalt grouping in individuals with ASD, particularly with respect to similarity grouping, might be based on less efficient allocation of neuronal processing to task demands.
